# Smart filtering of phase residues in noisy wrapped holograms

**DOI:** 10.1038/s41598-020-74131-8

**Published:** 2020-10-12

**Authors:** Behnam Tayebi, Farnaz Sharif, Jae-Ho Han

**Affiliations:** 1 Neuroscience Institute, New York University Langone Health, New York, NY 10016 USA; 2 Department of Ophthalmology, New York University Langone Health, New York, NY 10016 USA; 3 Department of Ophthalmology, Boston Children’s Hospital, Harvard Medical School, Boston, MA 02115 USA; 4 Department of Brain and Cognitive Engineering, Korea University, Seoul, 02841 South Korea; 5 Department of Artificial Intelligence, Korea University, Seoul, 02841 South Korea

**Keywords:** Optical techniques, Microscopy, Optical imaging

## Abstract

Phase unwrapping is one of the major challenges in multiple branches of science that extract three-dimensional information of objects from wrapped signals. In several applications, it is important to extract the unwrapped information with minimal signal resolution degradation. However, most of the denoising techniques for unwrapping are designed to operate on the entire phase map to remove a limited number of phase residues, and therefore they significantly degrade critical information contained in the image. In this paper, we present a novel, smart, and automatic filtering technique for locally minimizing the number of phase residues in noisy wrapped holograms, based on the phasor average filtering (PAF) of patches around each residue point. Both patch sizes and PAF filters are increased in an iterative algorithm to minimize the number of residues and locally restrict the artifacts caused by filtering to the pixels around the residue pixels. Then, the improved wrapped phase can be unwrapped using a simple phase unwrapping technique. The feasibility of our method is confirmed by filtering, unwrapping, and enhancing the quality of a noisy hologram of neurons; the intensity distribution of the spatial frequencies demonstrates a 40-fold improvement, with respect to previous techniques, in preserving the higher frequencies.

## Introduction

Denoising data is a critical process for reducing unwanted information in several branches of science^1,2^. However, in most cases, conventional denoising techniques degrade the quality of the desired information and thus can cause errors in the extraction of the real information. Biomedical data include critical information that must be accurately interpreted. Digital holographic microscopy is a non-invasive, wide-field, high-resolution interferometric biomedical imaging tool to study cell biology^3–8^. However, the noise level of the hologram, caused by speckles, sensor shot noise, and other sources, can destroy the object information. Although several noise-reduction techniques have been developed^9,10^, most of these techniques decrease the resolution. Recently, non-local denoising techniques that preserve the fine features of images have been developed^11–13^; however, in attempts to denoise holograms that are wrapped, these approaches have failed because of the periodicity of the phase map with a sawtooth shape, and hence they can only be applied to unwrapped phases. To overcome such problems, several unwrapping techniques have been developed in the last few decades, and these can be categorized as either physical or numerical methods. Physical and optical phase unwrapping methods mainly deal with the phase jumps by employing multiple wavelengths^14–17^, multiple illuminations^18,19^, or gradient phase techniques^20^. Multiple-wavelength phase unwrapping is based on the comparison of phase maps recorded using different wavelengths. This technique relies on the fact that the discontinuities in the phases acquired using different wavelengths occur at different locations. Multiple-illumination phase unwrapping works by reducing the recorded phase difference of the different illumination angles to less than 2π to avoid phase wrapping. The gradient method records the phase difference between two neighboring pixels; therefore, the recorded phase difference is mostly less than 2π and there is no wrapping in the data^20^. These techniques require additional physical components, increasing their complexity. Furthermore, the noise level of the system increases with the power of the unwrapping or dynamic range.

In contrast, numerical techniques for phase unwrapping have become more popular with the improvement in computer processing speed and the reduction in computational costs. Numerical techniques are mainly based on either directly unwrapping^21–27^ or filtering^28–35^ the wrapped phase. Examples of the first of these types of numerical techniques have been developed using global algorithms^23,24^, path-following algorithms^25^, and region-based algorithms^26,27^. Although they have been successful in various fields of science and industry, increase of residue noise decreases the effectiveness of these techniques.

The second type of numerical phase unwrapping method is based on decreasing the number of phase residues in a wrapped phase by filtering. Because of the sawtooth shape of the wrapped phase, conventional filters such as low-pass filters destroy the phase jumps. Several filters have been designed for reducing the noise in a wrapped phase. These include the fringe smoothing approach^28^, local fringe frequency estimation^29^, regularized phase-tracking method^30^, windowed Fourier filtering (WFF)^31^, windowed Fourier filtering and quality-guiding (WFF-QG)^32^, windowed Fourier ridges method^33^, Gabor filter local frequency^34^, and sine/cosine average filter (SCAF)^35^. These techniques reduce the noise level of highly wrapped and noisy phases. However, when the noise level increases, strong filters should be employed, which increases the numbers of artifacts in the filtered image. These filters are typically applied to all the pixels in a wrapped phase; therefore, even for a limited number of residues, artifacts are added to all the pixels. Furthermore, they cannot distinguish between residue noise and high-resolution image information; therefore, these filters destroy the image resolution to solve the wrapping problem. Degrading the resolution is costly because the high numerical aperture objective lens, which is typically the most important feature in the microscope, is rendered useless by applying such a filter.

Here, we present a novel, iterative, adaptive filter to locally reduce the number of phase residues by employing an appropriate filter size (window) in a minimum area (patch) only, around the residue. In contrast with the previous filters designed for unwrapping, this technique only applies filtering to the phase residues; by employing an iterative phasor average filtering (PAF) algorithm, it locally removes the phase residues. Both patch size and filter power are increased, step-by-step, from the minimum possible sizes to ensure that the minimum patch size and filter power necessary are used to remove the phase residues. First, keeping the patch size constant, the filter power is gradually increased, and the iteration is complete when no residue is detected. If, after employing the maximum filter power, the residues are not removed, the patch size is increased, and the process of increasing the filter power is repeated. The entire process continues until the residues are removed or the maximum filter power and the maximum patch size have been selected. By employing these steps, the PAF artifacts, which increase with the power of the filter, can be locally restricted. As a result, the wrapped phase with a minimum number of residues is obtained; in this way, a simpler phase unwrapping process is used to address the discontinuities. Simulated and experimental results presented in the following sections of this report demonstrate the feasibility of the technique for phase unwrapping and reducing the noise level of images with minimal artifacts. Surprisingly, for the cases in which strong PAF fails to solve the wrapping problem, we show that smart phasor average filtering (SPAF) unwraps the phase while preserving image resolution.

## Methods

For a two-dimensional (2D) noisy wrapped signal (*φ*), the noisy measured signal can be expressed as

1 $$N(\varphi ) = \varphi_{N} + \varphi_{R} ,$$

where *φ*_*N*_ and *φ*_*R*_ denote the phase of pixels without and with phase residue noise, respectively. If an ordinary filtering technique were to be applied to both the terms in (1), most of the higher frequencies of the image would be removed. To elucidate this point, before presenting the proposed technique, we explain how a classical filter operates on a wrapped phase.

### Phasor average filtering (PAF)

The object wavefront can be captured by both in-line and off-axis interferometry; however, off-axis arrangements have the advantages of allowing single-exposure imaging and spatial frequency multiplexing^36,37^. The intensity captured by such a system can be expressed as

2 $$I_{sensor} = \left| {E_{R} (x,y)} \right|^{2} + \left| {E_{S} (x,y)} \right|^{2} + 2E_{R}^{*} (x,y).E_{S} (x,y)\, \cdot \cos (q \cdot x + \varphi_{S} (x,y) - \varphi_{R} (x,y)),$$

where *E*_*R*_, *E*_*S*_, *φ*_*R*_, and *φ*_*S*_ indicate the amplitudes and phases of the reference and sample arms, respectively. Further, *q* is the spatial frequency of the fringes. By taking a Fourier transform of the intensity at the sensor plane and performing first-order filtering, we obtain

3 $$FT(E_{S} ) = [2FT\{ E_{R}^{*} (x,y) \cdot E_{S} (x,y)\} \otimes FT\{ \exp (q \cdot x\, + \varphi_{S} (x,y) - \varphi_{R} (x,y))\} ] \cdot f_{NA} ,$$

where *f*_*NA*_ is an appropriate filter that preserves the highest frequencies of the system. Taking the inverse Fourier transform of (2) and ignoring the filtering effect, we can extract the complex amplitude as

4 $$E_{O} = \frac{{E_{S} (x,y)}}{{E_{b} (x,y)}} \cdot \exp (\varphi (x,y)),$$

where *E*_*b*_ is the amplitude of the background image and *φ* denotes the phase difference related for the object. The wrapped phase difference can be obtained by taking the phase of the exponential function in (4). Thus, it includes sawtooth jumps at the wrapped positions; therefore, in the presence of noise, the unwrapping process is difficult. Furthermore, noise filtering by conventional methods may change and smooth the jump frequencies, which are vital for the accurate performance of unwrapping techniques. In contrast, the exponential phase or phasor in (4) does not have sawtooth jumps, and local filters such as low-pass filters can therefore be employed to reduce the phasor noise. The filtered phase can be expressed as

5 $$\psi = \ell [\varphi ] = \arg \left\{ {L\left[ {\exp (i\varphi )} \right]} \right\},$$

where *L* is the median or low-pass filter operator. Techniques such as SCAF and WFF have been developed based on filtering (5), to reduce the number of phase residues. However, when the filters are employed for all the pixels of the image, the number of artifacts in the filtered image increases.

### Smart phasor average filtering (SPAF)

**Figure 1:**
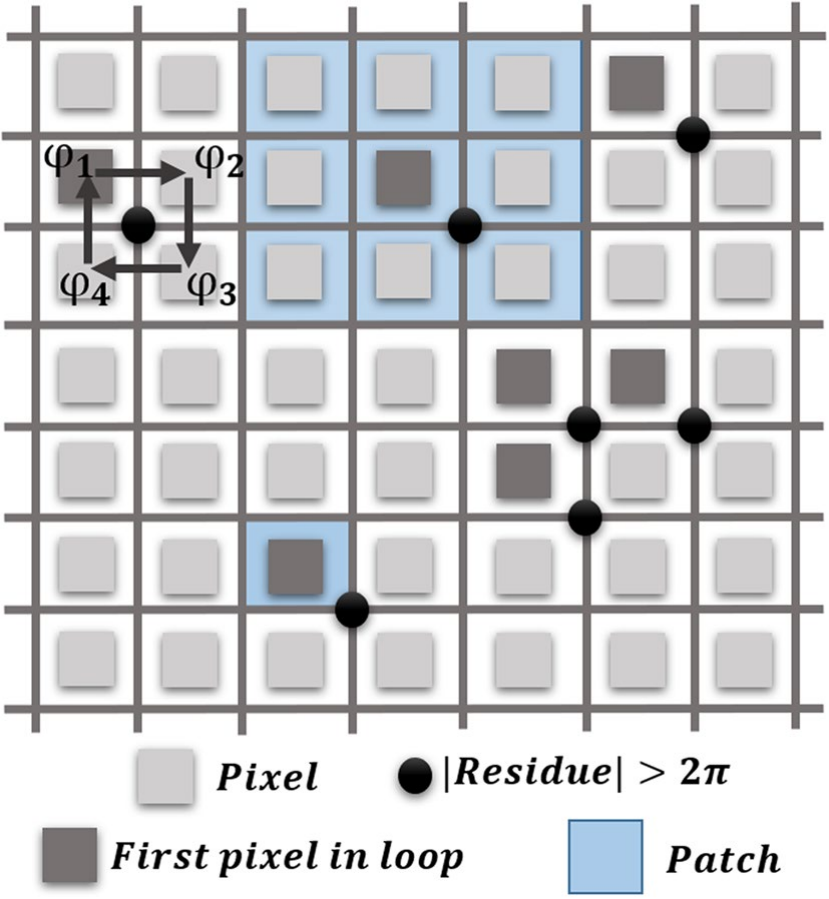
Schematic of a noisy wrapped signal; the light gray, dark gray, and blue squares represent the simple pixels, first pixels in a residue loop, and patches, respectively. The black circles denote the residues of four pixels.

**Figure 2:**
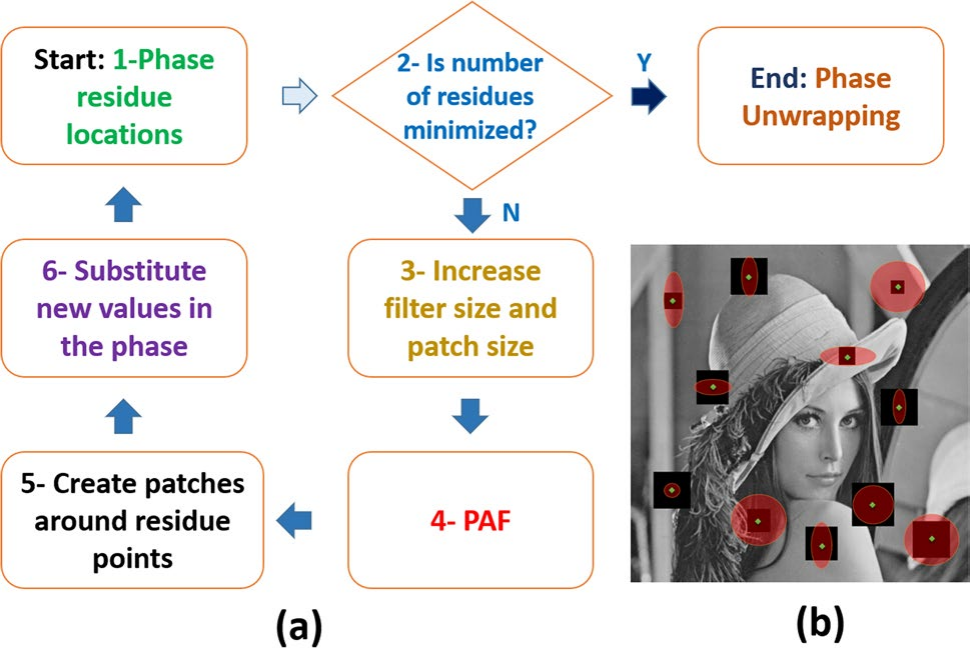
(**a**) Block diagram of principles of the smart phasor average filtering (SPAF) technique. (**b**) Schematic of patch sizes and PAF filters. The green points represent the phase residues that spread around the image. The black squares are the patches around the phase residues, and the patch sizes increase from unity to the same maximum value in both directions. Further, the red circles represent the size of the PAF in each step; the size of the filter can take different values in the x and y-directions.

Figure 1 shows a schematic of the pixels of a noisy wrapped signal; the light gray, dark gray, and blue squares represent the simple pixels, first pixels in a residue loop (FPRs), and patches, respectively. The black circles denote the non-zero residues of four pixels. The residue for any 2 × 2 closed-loop path {*P*} can be calculated as

6 $$\sum\limits_{p = 1}^{4} {\nabla \varphi_{p} } = 2\pi n,$$

where $p \in \{ P\}$, $\nabla$ is a gradient operator, and *n* is an integer. From the residue map, the locations of the normal and residue noises can be separated with two binary matrixes as

7 $$U = U_{N} + U_{R} ,$$

where *U* is an all-ones matrix. *U*_*N*_ and *U*_*R*_ are the binary and complementary matrices that determine the locations of the pixels with the normal and residue noise, respectively. In order to simplify the calculation, only the FPR is considered in *U*_*R*_. Furthermore, because the residues may be caused by other pixels in the residue loop, and because the filtering of any pixels may change the residue map, a patch operator is acted upon (7); therefore, it should be rewritten as

8 $$U = [1 - P_{wp} (U_{R} )] + P_{wp} (U_{R} )$$

where *P* is the patch operator that takes the pixels around each FPR within a specific window (*wp*). Therefore, *P*_*wp*_ (*U*_*R*_) is a binary matrix that determines the locations of the pixels that should be filtered at each step. The pixels are separated into two groups, unfiltered (first term) and filtered (second term) pixels. Therefore, the phase in each step can be expressed as

9 $$\varphi^{m} = [1 - P_{{wp_{m} }} (U_{R}^{m} )]\, \cdot \,\varphi^{m - 1} + P_{{wp_{m} }} (U_{R}^{m} )\, \cdot \ell_{{wf_{m} }} [\varphi^{m - 1} ],$$

where *ℓ*_*wf*_ is the median phasor-filter operator with window size *wf* that operates on the phase at the *m*^th^ step and *wf* is the number of the neighboring pixels in the median filter. The pixels in the first term of (9) are not changed in this process and median filtering is only employed for the second group. Equation (10) can be applied via the following steps: first, the locations of the phase residues are obtained. In the second step, if the number of the residues is less than the desired minimum value (zero), the process is completed and a simple phase-unwrapping technique is employed. If not, in the third step, the window size of the median phasor filter is increased. It should be emphasized that if the size of the patches is increased before the window size is increased, the amount of artifacts may be further increased; for this reason, we first increase the window size, and then the patch size. In the fourth step, the new phasor filter is operated on the phase map. In the fifth step, according to the selected patch size of the step, an all-ones sub-matrix around each residue (black squares in Fig. 2b) is created; this matrix is the binary patch matrix, *P*_*wp*_(*U*_*R*_). Therefore, *P*_*wp*_(*U*_*R*_) is a binary matrix that is one only around the residues. In the sixth step, only the values of this new filtered phase in the patches around the residue points are substituted into the previous iteration phase map the second term in (9), and the value of the pixels outside of this binary matrix is constant (the first term in (9)). Next, the phase residues of the modified phase map are calculated and, if the number of residues has not reached the minimum, these steps are repeated. It is important to calculate phase residue location in each iteration of the algorithm because the position of the residues can be shifted between neighboring pixels during filtering. The loop should be terminated when the phase map is free of residues, but it can be terminated if the number of residues is minimized and cannot be lowered further on a reasonable timescale. The obtained noisy wrapped phase is locally filtered and hence the smallest phase features are preserved. When this condition is satisfied, the phase should be unwrapped; depending on the remaining residues, the filtered phase map can be unwrapped by a simple or complex unwrapping technique. In this study, we employed the simple one-dimensional phase unwrapping algorithm in MATLAB to address the ambiguities in the phase map. However, if the combination of SPAF and simple 1D unwrapping technique fails to unwrap the phase, we can combine it with 2D unwrapping methods such as quality guided or minimum network flow unwrapping methods. In that condition, the algorithm becomes more robust to noise.

Figure 2a outlines the principles of the technique in a block diagram. Figure 2b presents the Lena image as an example image employed to illustrate the schematic of the patch sizes and PAF filters. The green points represent the phase residues (FPR) that spread around the image. The black squares in Fig. 2b are the patches around the phase residues, and the patch sizes increase from unity to the same maximum value in both directions (to reduce computational cost). Further, the red circles in Fig. 2b represent the PAF size for each step; the PAF size of the filter can take different values in the *x* and *y* directions.

### Simulation methods

Simulations were performed on a generated Gaussian phase with a height of 30 rad in an image of 400 × 600 pixels, with added speckle noise and Gaussian white noise with a variance of 2 and signal-to-noise ratio of 6, respectively. A noise-free ideal Lena image was also studied. Classical techniques applied for comparison included 1D unwrapping and 2D least-squares cosine transform (LSCT)^38^, quality guided (QG)^39^, and 2D minimum network flow (MNF)^40^ unwrapping techniques, as well as PAF and WFF. SPAF was performed according to the schematic of Fig. 2 with a patch size between 2 and 15 pixels (depending on the residue noises).

### Experimental methods

We performed experiments using multiple-wavelength diffraction phase microscopy (MW-DPM)^8,15^, acquiring only the hologram of a 473-nm diode laser. The magnification and numerical aperture of the objective lens were selected as 40 and 0.95, respectively; hence, the lateral resolution of the imaging system is about 820 nm. The total magnification of the system was selected as 60. The camera pixel size was 7.4 µm with a frame rate of 110 frames per second. We used a cropped demultiplexing algorithm to extract the phase from modulated signal. The phase of one bead and eleven neuronal cells with different morphologies were used as input. Three arbitrary images with the same pixel size are used to show the effect of filtering on high resolution images. The minimum filter size was selected to preserve the maximum possible amount of information on the neurons.

## Results

### Simulation results

**Figure 3:**
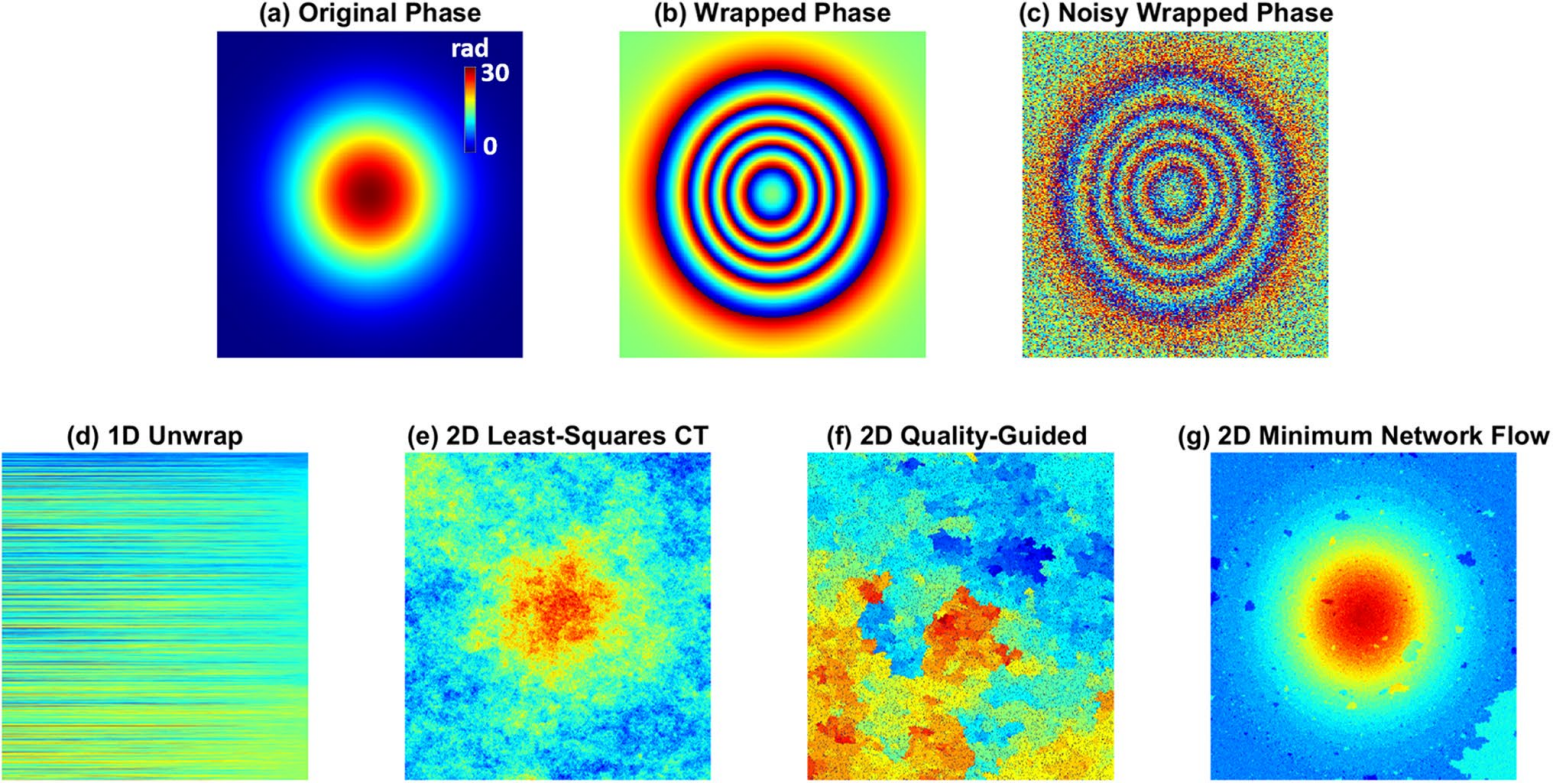
(**a**) Gaussian phase, (**b**) wrapped phase, and (**c**) noisy wrapped phase. Unwrapped phase using (**d**) one-dimensional (1D) unwrapping, (**e**) two-dimensional (2D) least-squares cosine transform, (**f**) 2D quality guided technique, and (**g**) 2D minimum network flow (MNF) techniques.

In order to study the ability of the proposed technique to minimize the phase residue in a wrapped phase map with minimal artifacts, and compare it with some classical techniques, we generated a Gaussian phase with a height of 30 rad in an image of 400 × 600 pixels. The phase is shown in Fig. 3a and the corresponding wrapped phase is shown in Fig. 3b. Figure 3c indicates that speckle noise and Gaussian white noise with a variance of 2 and signal-to-noise ratio of 6, respectively, are added to the wrapped phase map. Figure 3d demonstrates that the 1D unwrapping techniques fail in addressing the wrapping problem because of the high number of residues. Figures 3e–g show the phases as unwrapped by the 2D LSCT, 2D QG, and 2D MNF unwrapping techniques, respectively. As can be seen, the 2D MNF algorithm can resolve the unwrapping problem, producing a higher-quality unwrapped phase.

**Figure 4:**
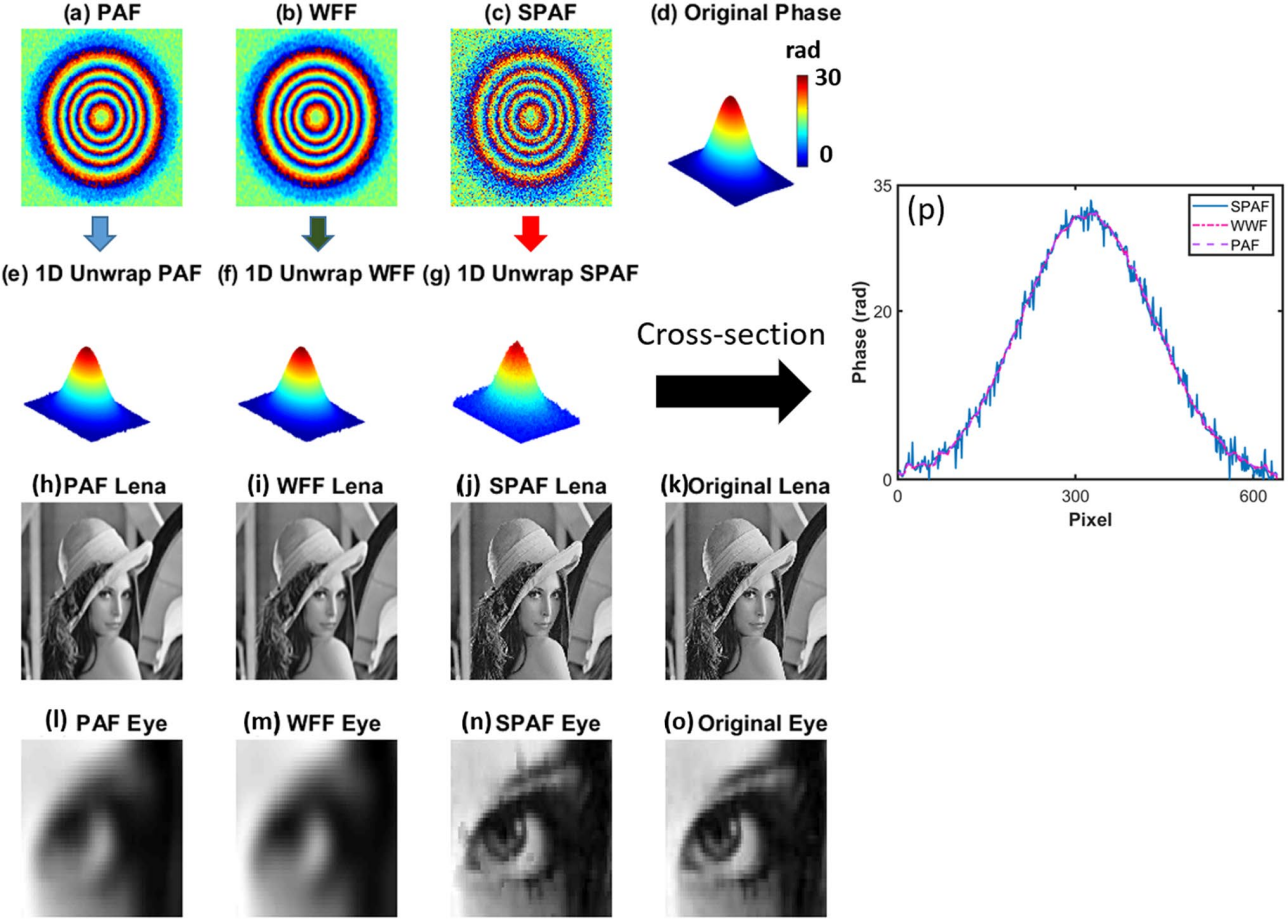
Filtered wrapped phase using (**a**) PAF, (**b**) windowed Fourier filtering (WFF), and (**c**) SPAF to minimize the number of phase residues. (**d**) Original Gaussian phase in three dimensions without noise. 1D unwrapped phases filtered by (**e**) PAF, (**f**) WFF, and (**g**) SPAF. Corresponding Lena image filtered by (**h**) PAF, (**i**) WFF, and (**j**) SPAF. (**k**) Original image. Magnified eye for (**l**) PAF, (**m**) WFF, (**n**) SPAF, (**o**) original image. (**p**) Cross-sections from the middles of (**e**–**g**).

However, the unwrapping time in this case is longer than that for the LSCT method and depends on the number of residues. Figure 4a,b present the filtered wrapped phase of Fig. 3c obtained using PAF and WFF, respectively, with minimal filtering effects to minimize the number of phase residues. Figure 4c illustrates the results of SPAF to minimize the number of phase residues. Using this technique, the resultant noise level is still high; however, the residues have been removed. Figure 4d illustrates the original phase without noise in a three-dimensional (3D) plot. Figures 4e–g show the unwrapped phases filtered by PAF, WFF, and SPAF, respectively. All the phases were unwrapped with the same 1D unwrapping (U) technique. The phase in Fig. 4g is noisier than those in Fig. 4e,f; however, there is a significant difference between these figures that makes the results of SPAF more appropriate for high-resolution microscopy. In order to clarify this point and investigate the loss of small features from an image, all the processes that were performed on the phase shown in Fig. 3c to obtain Fig. 4e–g were applied to a noise-free ideal Lena image (of the same size).

We used this noise-free image because we wanted to investigate the effect of each filter on an image with higher spatial frequencies. These processes were selected to provide us with comparable results on the effect of each filter on a standard high-resolution image. The technique can also be applied to a noisy image; however, because the initial frequencies in the noisy image are already corrupted, the comparison would be more difficult. In order to apply the filters, the Lena image was normalized to avoid the wrapping problem, enabling the filters to operate on it.

Figure 4h–j display the Lena image after filtering by PAF, WFF, and SPAF, respectively; the original Lena image is shown in Fig. 4k. Although most of the phase residues were removed by a filter with a small size to produce the phase shown in Fig. 4g, to completely remove some phase residues, a large window is required for this phase (Fig. 4g). Therefore, the first two filters globally blur the Lena image, as expected; however, smart filtering blurs the image locally. Thus, SPAF could be more appropriate for preserving the small features of the image. Figure 4l–o are close-up views of the eye in the Lena images displayed in Fig. 4h–k, respectively. As can be seen in Fig. 4n, most of the fine features are preserved as a result of filtering a limited number of pixels when using the SPAF technique. Figure 4p is a cross-section line through the middle of Fig. 4e–g and compares the variation of these three phases. As can be seen in Fig. 4p, the variation of SPAF is much larger than PAF and WFF, which demonstrates that SPAF has less image distortion compared to other methods.

### Experimental results

**Figure 5:**
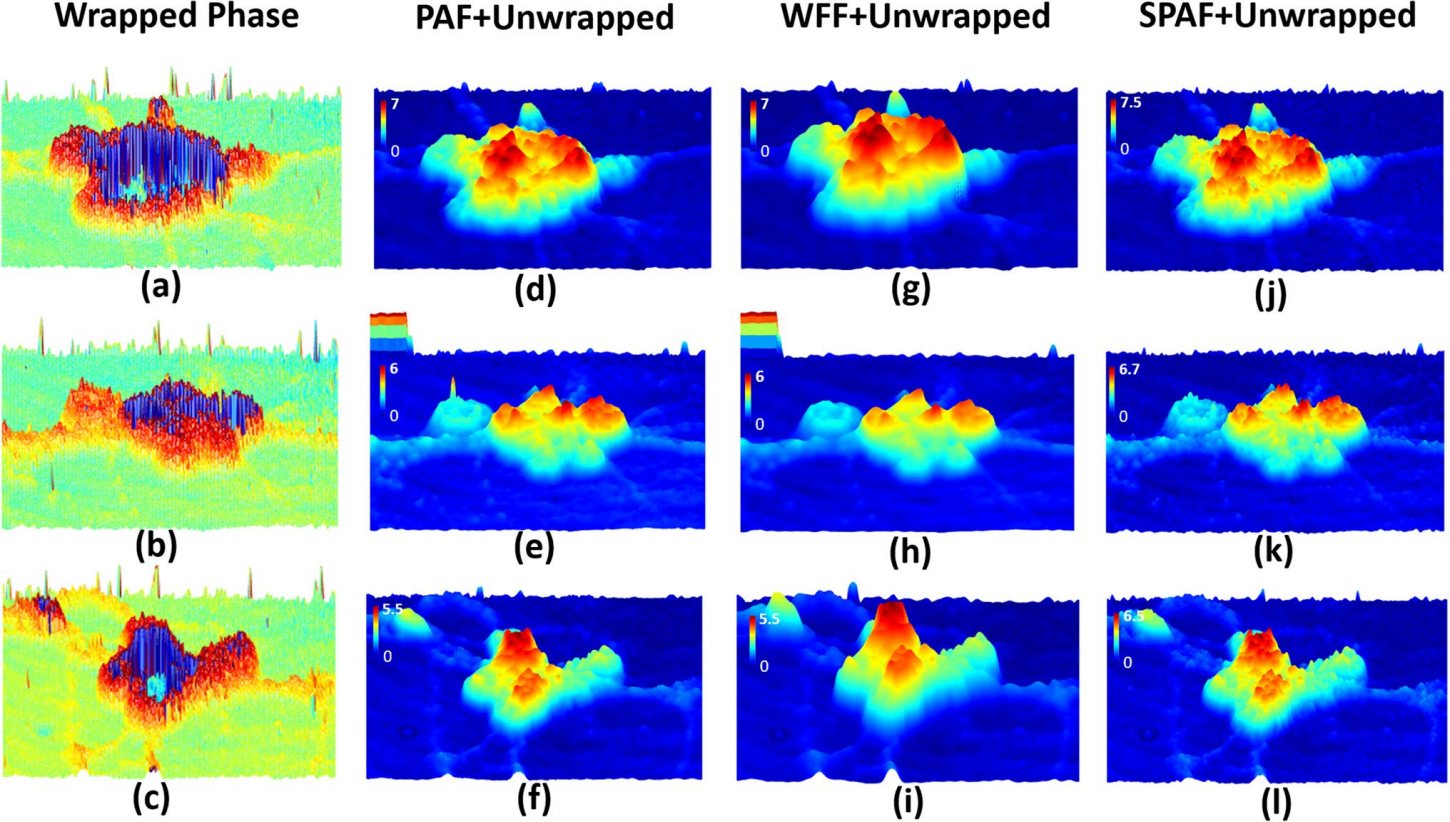
(**a**–**c**) are original noisy wrapped phase of three different neurons. Unwrapped phases after filtering by (**d**–**f**) PAF, (**g**–**i**) WFF, and (**j**–**l**) SPAF.

The noisy wrapped phases of three neuronal cells acquired using MW-DPM are presented in Fig. 5a–c. Figure 5d–f show the PAF-filtered and 1D-unwrapped images of Fig. 5a–c, respectively. The minimum filter size was selected to preserve the maximum possible amount of information on the neurons. However, as is clear in Fig. 5e, a larger filter size should be employed to successfully unwrap the edge of the image. Figure 5g–i show the WFF-filtered, 1D-unwrapped images of Fig. 5a–c, respectively. As can be observed, the quality of the images is considerably reduced. Figure 5j–l illustrate the SPAF-filtered, 1D-unwrapped images of Fig. 5a–c, respectively. In these cases, the holograms were successfully unwrapped. Furthermore, most of the details of the image were not degraded by the SPAF process. In order to clarify the issue and further examine the losses in the small features of an image, all the processes that were performed on the phases in Fig. 5a–c to obtain Fig. 5d–l were applied to three arbitrary images (Supplementary Fig. S1a–c). PAF and WFF, with the same filters (same locations and same sizes) that were employed to obtain Fig. 5d–i, were applied to the three arbitrary images, and the results are shown in Supplementary Fig. S1d–i, respectively. The results show that PAF and WFF blur most of the details of the images. Furthermore, Fig. S1j–l show the effect of employing the SPAF filter to the corresponding Fig. 5j–l, respectively. In order to evaluate the filtering effects more clearly, for each image, we expanded an area within it, outlined in red, which is displayed as an overlaid image in each case. These expanded views of the image details allow us to confirm that the proposed technique filters the phase residues and minimizes blurring in noisy holograms. The SPAF technique, therefore, preserves more of the critical image information.

**Figure 6:**
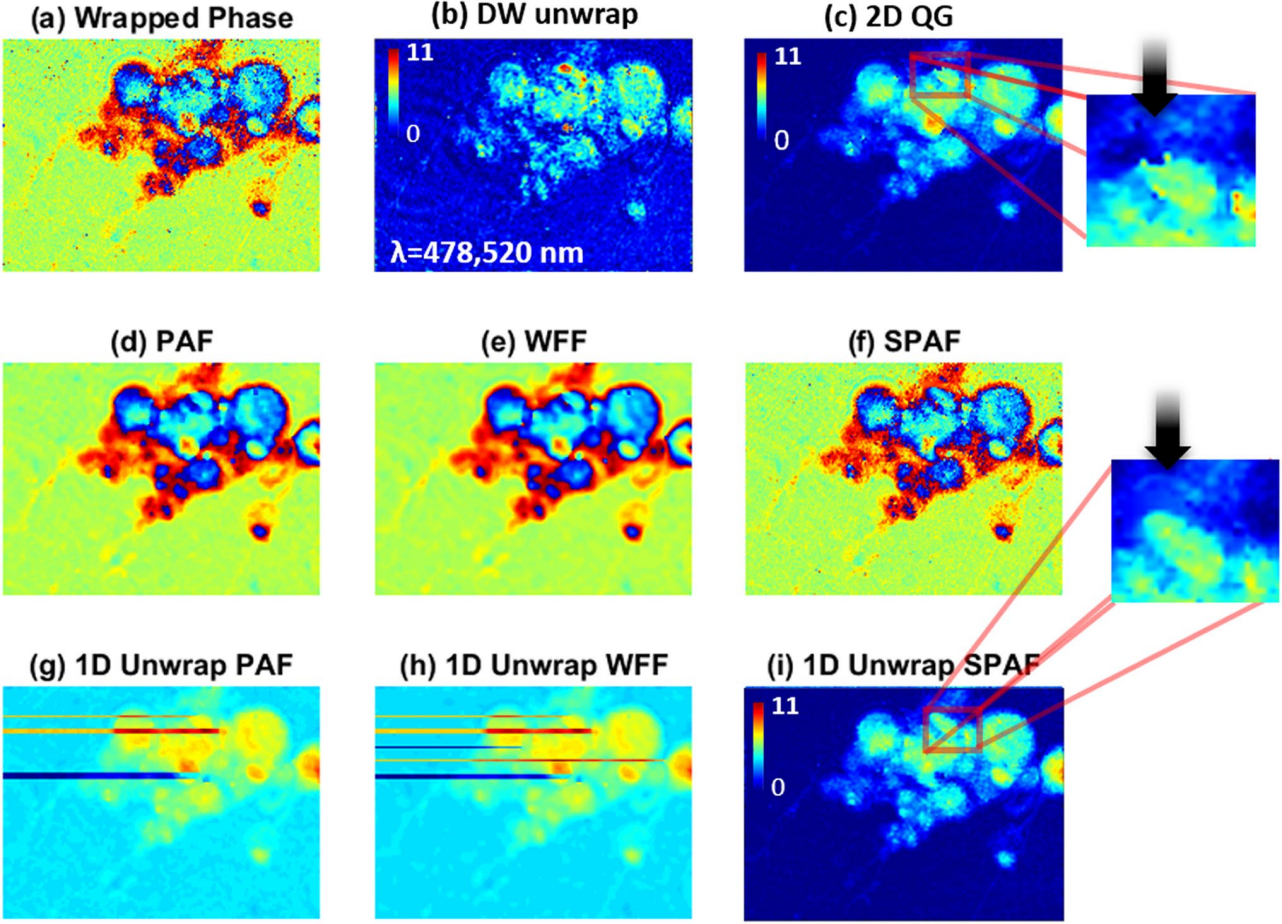
(**a**) Original wrapped phase of neurons. Unwrapped phase by (**b**) Dual-wavelength (DW) and (**c**) 2D quality guided (QG) techniques. Filtered phase by (**d**) PAF, (**e**) WFF, and (**f**) SPAF. (**g**–**i**) Corresponding 1D unwrapped phases of (**d**–**f**), respectively. Color bars show the phase in radian.

Figure 6 compares the results of applying the dual wavelength (DW), 2D QG, PAF, WFF, and SPAF techniques to another noisy hologram with a higher number of phase residues. Figure 6a shows the original wrapped hologram, and the results of the DW and 2D QG unwrapping procedures are presented in Fig. 6b,c, respectively. It appears that both DW (hardware) and 2D QG (numerical) unwrapping techniques were successful. However, in the DW technique, the noise level is amplified by the ratio of the synthetic wavelength to the original wavelength of the phase. In this case we recorded an additional phase with 520 nm so the synthetic wavelength is 5.9 µm and the ratio (Ʌ/λ = 5.9/0.478) is around 12, and thus both dynamic range and noise level are increased 12 times. In the DW technique the noise level can be reduced using a hierarchical technique if the phase’s signal to noise ratio (SNR) follows the conditions that are mentioned in reference^41^. However, because we intentionally select noisy phases for investigation in our method, it does not fulfil the hierarchical noise reduction technique’s conditions. Therefore, using the above wavelengths, the results of the DW technique are too noisy compared to numerical phase unwrapping techniques.

Figure 6d–f show the filtered phase produced by using the PAF, WFF, and SPAF algorithms, respectively. Figure 6g–i show the corresponding 1D unwrapped phases corresponding to those in Fig. 6d–f, respectively. We selected the maximum size of the patches and PAF to be about five times the resolution of the microscope to obtain the result shown in Fig. 6i. It should be noted that this filter was only employed for a limited set of pixels. It is clear from these results that the proposed technique is more robust to the residue noise than PAF or WFF. In this letter, we do not compare the results of our proposal to those of the 2D phase unwrapping techniques; however, the expanded view shown in Fig. 6c reveals that some phase residues cannot be easily unwrapped by the 2D QG unwrapping technique. As can be seen in the expanded view in Fig. 6i, one of the effects of reducing the number of phase residues is avoiding incorrect phase jumps in the final unwrapped phase map. Furthermore, the processing time for QG is about 3 s, almost one order of magnitude slower than SPAF.

In order to evaluate the performance of the algorithm on additional experimental example images, we applied the proposed technique to eight more neuronal phases with different morphologies. Supplementary Figs. S2 and S3 show the results for the three different techniques; it can be observed that WFF is more successful than PAF. Furthermore, it can be seen that SPAF successfully filtered the phase residues of all the phases and the unwrapping has no error. However, an interesting result is the fact that, in the most of the images, when the PAF method, even with strong filtering, cannot remove the phase residues (Supplementary Figs. S2d,S3), SPAF, which is based on local PAF, can easily remove the phase residues. The reasons for this result could originate from the changing of the location and intensity of the residues in each step in SPAF, compared to the constant location used throughout the PAF procedure.

Supplementary Table SI lists the computational times for operating the SPAF algorithm using an Intel core i7-3770 CPU @ 3.4 GHZ and 8.00 GB of RAM; all code was written with MATLAB and no GPU was employed. However, implementing the algorithm using CUDA can significantly reduce the processing time^42^. It can be seen the computational time is not constant and depends on the sample, varying from 0.046 to 4.339 s. Although the speed of the algorithm is not the subject of this article, it should be mentioned that these computational times can be considered appropriate for noisy unwrapped phases.

## Discussion

**Figure 7:**
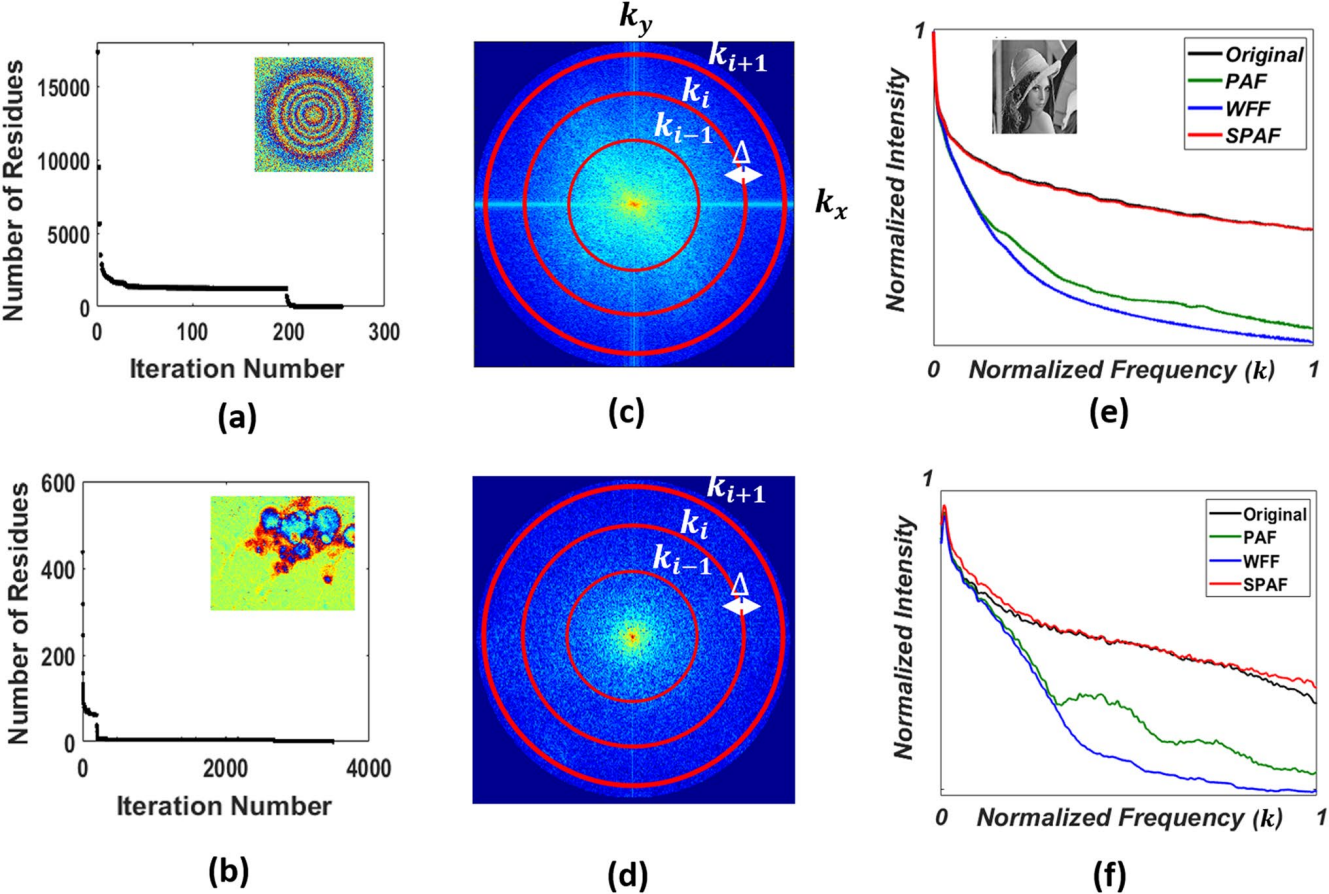
Number of residue points as a function of iterations for (**a**) simulation and (**b**) experimental results. Absolute value of the Fourier transform of the filtered image by SPAF for (**c**) Lena and (**d**) Neuron image. Normalized intensity distribution in the frequency domain as a function of the normalized absolute spatial frequency for (**e**) Lena and (**f**) Neuron image.

In this paper, we presented a smart technique to filter the phase residues in wrapped phase maps. It is demonstrated that it is not necessary to denoise all the pixels in the image to reduce the number of phase residues. The drawbacks of ordinary filters are locally minimized by applying them only to the locations of residues. Figure 7a,b illustrate the reduction in the number of phase residues for Fig. 4 and 6, respectively. As can be observed in the case of the simulation (Fig. 7a), the number of residues is considerably higher than that in Fig. 7b, which shows the data for the experimental image; however, the iteration number for Fig. 7a is much lower than that for Fig. 7b. This is because, in the simulation, we assumed normal Gaussian white noise and speckle noise; however, in nature, the noise and phase residues would be more complex. It can be observed that most of the residue points were removed in the initial steps; however, more residues can be removed by applying higher iteration numbers.

Another issue that should be discussed is the error in the filtered image. In order to quantify this type of error, we must first calculate the average relative square error (RSE) for each pixel in Fig. 4h–j. The RSE can be expressed as

10 $$RSE = \frac{{\left\| {\varphi - \varphi_{GT} } \right\|^{2} }}{{\left\| {\varphi_{GT} } \right\|^{2} }},$$

where *φ*_*GT*_ is the ground-truth phase. It should be mentioned that this error cannot be calculated for the neuronal image in Fig. 6, because there we have no ground-truth for it. The average of the RSE of the noiseless Lena images treated using PAF, WFF, and SPAF are 6.99, 6.01, and 0.68%, respectively.

These numbers represent the average error in the filtering of each pixel; however, to determine the changes in the small features of the image, we studied the intensity at each spatial frequency. Figure 7c,d present the natural logarithm of the Fourier transforms of the images in Figs. 4j, 6i, obtained using

11 $$I(\overrightarrow {k} ) = \left\| {\iint {e^{{i\varphi (\overrightarrow {x} )}} }e^{{( - i\overrightarrow {k} .\overrightarrow {x} )}} \,d\overrightarrow {x} } \right\|.$$

The absolute spatial frequency *k* can be calculated using

12 $$k_{i} = \sqrt {k_{{x_{i} }}^{2} + k_{{y_{i} }}^{2} } ,$$

where *k*_*xi*_ and *k*_*yi*_ are the *x* and *y* components of the spatial frequency at point *i*. The average intensity of the points with the same spatial frequency *k* as the red circles shown in Fig. 7c can be expressed as

13 $$\overline{I} (k_{i} ) = \frac{1}{{2\pi k_{i} \Delta }}\int_{{k_{i} - \frac{\Delta }{2}}}^{{k_{i} + \frac{\Delta }{2}}} {\,\ln [I(k)} \,]\,dk,$$

where Δ is the thickness of each circle. Figure 7e presents the normalized intensity distribution in the frequency domain as a function of the normalized absolute spatial frequency. As can be seen, the SPAF technique preserves the higher frequencies of the image. The average RSE values for the normalized intensity distributions of the Lena images in the frequency domain (Fig. 7e) obtained using PAF, WFF, and SPAF are 57.51, 80.00, and 0.01%, respectively. The average RSE of the normalized intensity distribution of the neuron image, shown in Fig. 6a, in the frequency domain (Fig. 7f) obtained using PAF, WFF, and SPAF, all after 1D unwrapping, are 41.73, 75.64, and 1.04%, respectively, which proves the ability of SPAF to reduce the number of residues with minimal artifacts.

The RSE of the normalized intensity of the frequency distributions for different samples are given in Supplementary Table SI. It can be seen that PAF has reduced error levels with respect to WFF; however, it should be noted that PAF, in many cases, results in a failure to unwrap the phase. Finally, the RSE of SPAF is significantly less than those of both WFF and PAF.

Using our approach, the number of phase residues for simulated and experimental wrapped phases was reduced to zero, with minimal artifacts. The errors in the intensity distributions of the spatial frequencies of experimentally acquired images filtered by SPAF were significantly reduced; this demonstrates the ability of SPAF to preserve image object features. Our experimental results demonstrate that SPAF not only can preserve resolution, but it also can successfully unwrap the phases that even a strong PAF algorithm cannot unwrap. This technique may open a new avenue to smart filtering of wrapped phases of images in which small features are particularly significant.

## Supplementary information

Supplementary Information

## Data Availability

SPAF codes for simulation and experimental data are available on https://github.com/behnamty/SPAF.
