# Supplementary material for: Smart filtering of phase residues in noisy wrapped holograms
Source: Sci Rep. 2020 Oct 12;10:16965. doi: 10.1038/s41598-020-74131-8 (PMC7552432; doi:10.1038/s41598-020-74131-8)
Supplement: Supplementary file 1 — Supplementary Information [file 41598_2020_74131_MOESM1_ESM.pdf]

Supplementary Figures and Tables

Smart Filtering of Phase Residues in Noisy Wrapped Holograms

Behnam Tayebi, Farnaz Sharif, and Jae-Ho Han

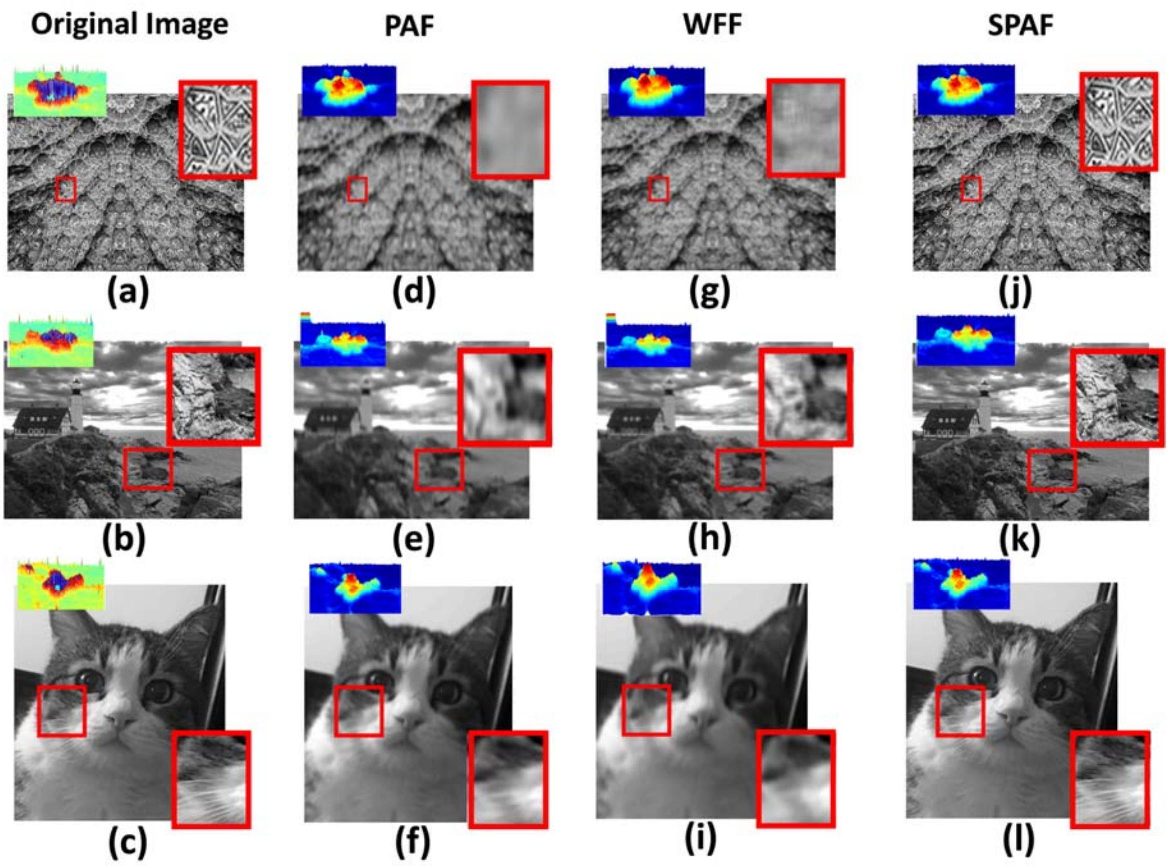

Figure S1. (a)-(c) are three original images. The images are processed by the same (d)-(f) PAF, (g)-(i) WFF, and (j)-(l) SPAF as Fig.5.

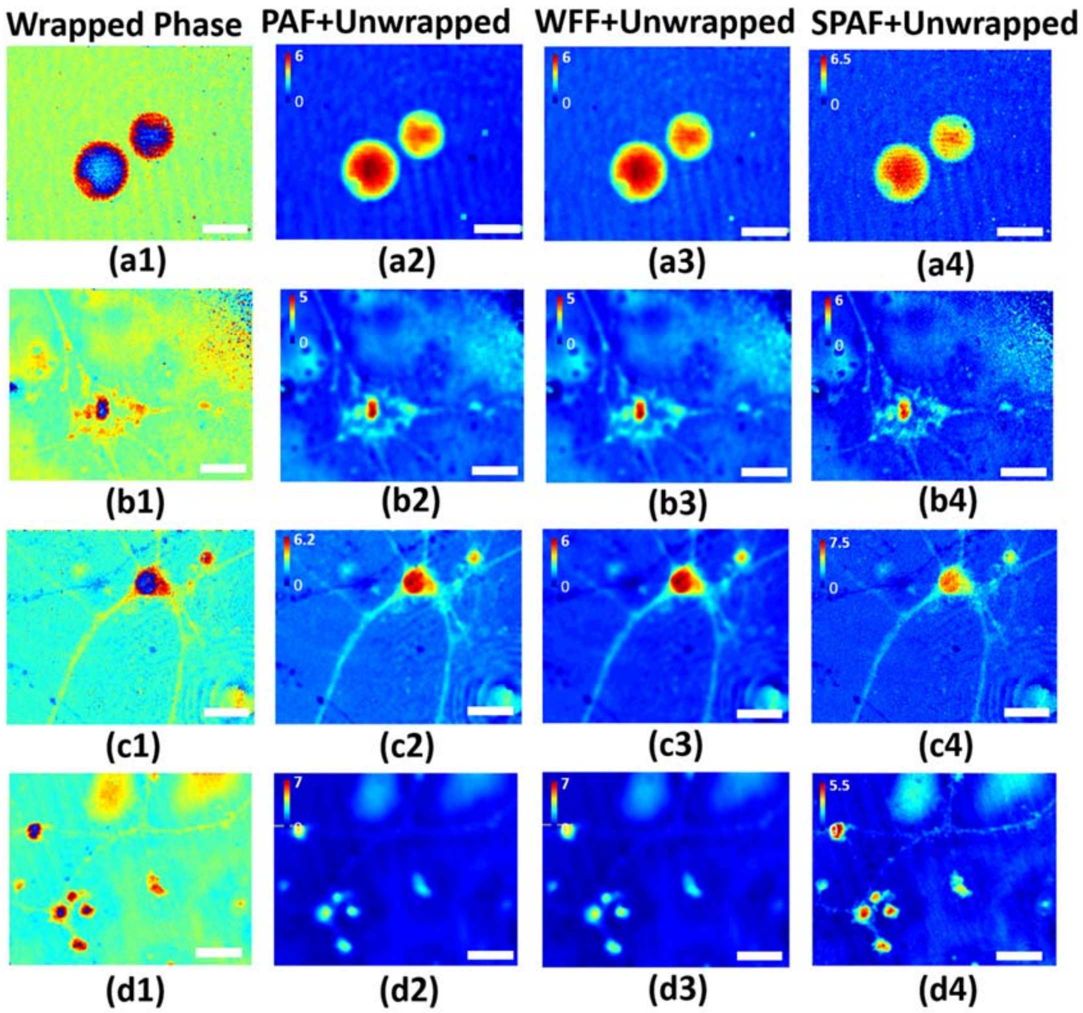

Figure S2. Original wrapped phase (a1, b1, c1, d1). Unwrapped phase using PAF (a2, b2, c2, d2), WFF (a3, b3, c3, d3), and SPAF (a4, b4, c4, d4).

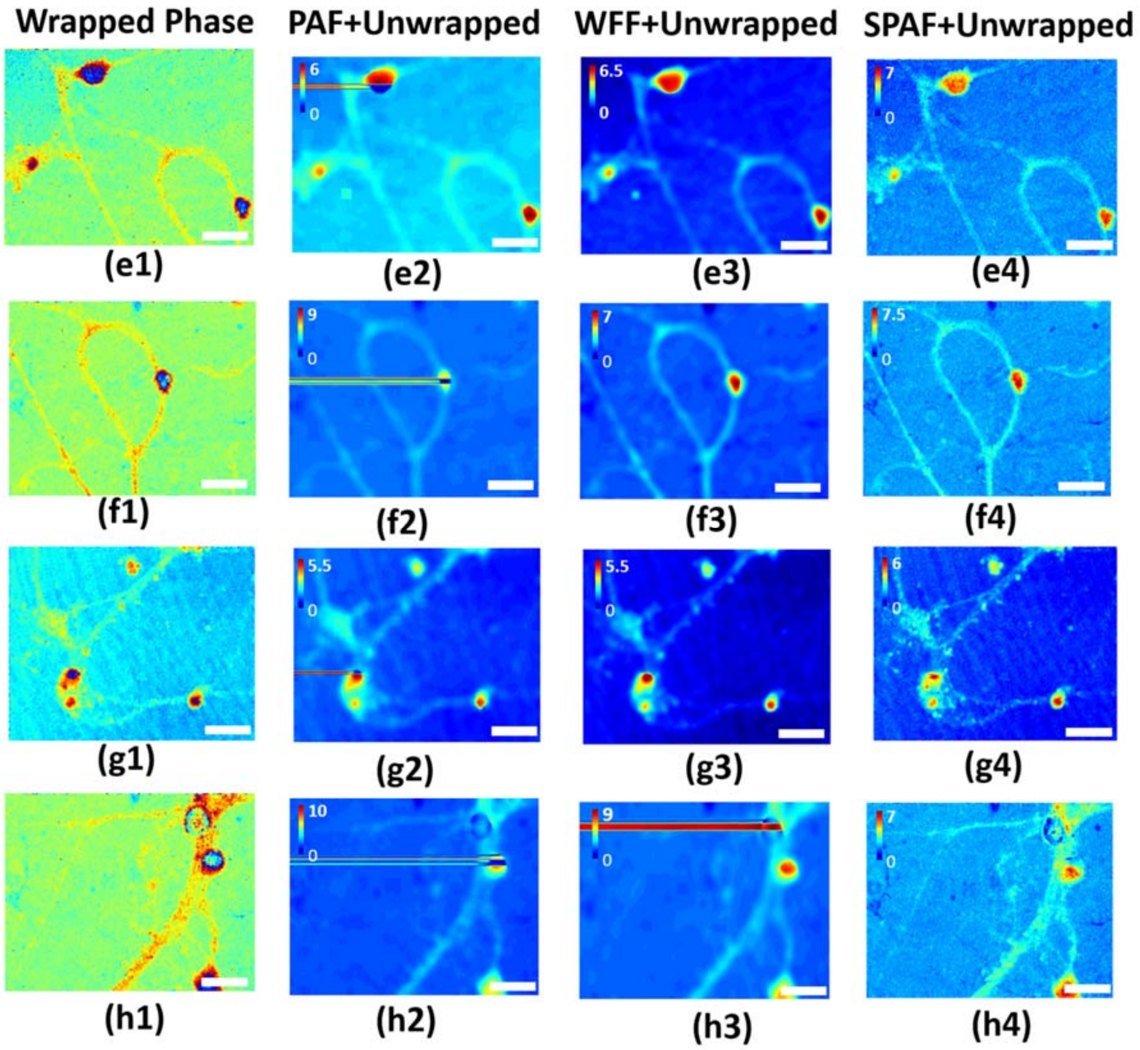

Figure S3. Original wrapped phase of neurons (e1, f1, g1, h1). Unwrapped phase using PAF (e2, f2, g2, h2), WFF (e3, f3, g3, h3), and SPAF (e4, f4, g4, h4).

Table SI. The computational time and RSE of different phases in Fig.S2 and S3 for PAF, WFF, and SPAF. Green and red show that the techniques are successful or unsuccessful in solving the wrapping problem, respectively.

**Image**	**Computational Time (s)**			**RSE**		
	PAF	***WFF***	***SPAF***	***PAF***	***WFF***	***SPAF***
a	0.015	1.000	0.046	26.1%	78.8%	1.24%
b	0.015	2.152	0.063	30.1%	60%	0.2%
c	0.013	1.341	0.120	16.5%	75.1%	0.5%
d	0.014	1.826	0.029	50.7%	77.5%	0.4%
e	0.017	2.203	0.047	46.4%	83.5%	1.6%
f	0.016	1.758	0.879	53.3%	85.4%	0.9%
g	0.015	1.154	2.057	42.3%	69.1%	1.2%
h	0.014	1.993	4.339	36.6%	75.7%	0.65%
